# Chemical Constituents Antioxidant and Anticholinesterasic Activity of *Tabernaemontana catharinensis*

**DOI:** 10.1155/2013/519858

**Published:** 2013-07-28

**Authors:** Carla Nicola, Mirian Salvador, Adriana Escalona Gower, Sidnei Moura, Sergio Echeverrigaray

**Affiliations:** ^1^Instituto de Biotecnologia, Universidade de Caxias do Sul, Francisco G. Vargas 1130, 95001-970 Caxias do Sul, RS, Brazil; ^2^Centro Tecnológico, Universidade de Caxias do Sul, Rua Francisco Getúlio Vargas 1130, Petrópolis, 95070-560 Caxias do Sul, RS, Brazil

## Abstract

The present work aimed to analyze the alkaloid content of the ethanolic extract of *Tabernaemontana catharinensis* (Apocynaceae family) and its fractions as well as to evaluate their antioxidant and anticholinesterasic activities. The analyses of the ethanolic extract of *T. catharinensis* by mass spectrometry allowed identifying the presence of the alkaloids 16-epi-affinine, coronaridine-hydroxyindolenine, voachalotine, voacristine-hydroxyindolenine, and 12-methoxy-n-methyl-voachalotine, as well as an alkaloid with *m/z* 385.21 whose spectrum suggests a derivative of voacristine or voacangine. The extract and its alkaloid rich fractions showed antioxidant activity, especially those that contain the alkaloid *m/z* 385.21 or 16-epi-affinine with DPPH scavenging activity (IC_50_) between 37.18 and 74.69 **μ**g/mL. Moreover, the extract and its fractions exhibited anticholinesterasic activity, particularly the fractions characterized by the presence of 12-methoxy-n-methyl-voachalotine, with IC_50_ = 2.1 to 2.5 **μ**g/mL. Fractions with 16-epi-affinine combined good antioxidant (IC_50_ = 65.59 to 74.69 **μ**g/mL) and anticholinesterasic (IC_50_ = 7.7 to 8.3 **μ**g/mL) activities, representing an option for further studies aimed at treating neurodegenerative diseases.

## 1. Introduction

Alzheimer's disease is a progressive neurodegenerative disorder related to genetic predisposition and characterized by the presence of neurofibrillary tangles, formation of extracellular deposition of *β*-amyloid peptide, oxidative stress, increased production of superoxide radicals, and reduced neurotransmitter levels [1, 2]. The treatments available to combat the symptoms of this condition are complex and include increasing acetylcholine levels in brain regions such as frontal cortex and hippocampus and possibly preventing neuronal degeneration with antioxidants [3].

Natural products derived from plants represent a viable alternative for discovering new potentially active substances.* Tabernaemontana catharinensis ADC *is a small tree of the *Apocynaceae *family currently found in Brazil, Argentina, Uruguay, Paraguay, and Bolivia [4]. The genus *Tabernaemontana *has evoked interest due to the important biologic activity of its extracts, particularly, antimicrobial [5, 6], anti-tumoral [7], antioxidant [8], anti-cholinesterasic [9], and anti-inflammatory [10] activities, most of which have been associated with indole alkaloids. Although several biological activities of *T. catharinensis* extracts have been reported, few substances with anticholinesterase activity able to minimize damage caused by oxidative stress have been described in *T. catharinensis*. The association of these properties may represent an alternative for the control of neurodegenerative disorders as Alzheimer's disease. 

 In view of the foregoing, the present work aimed to evaluate the content of the ethanolic extract obtained from the aerial parts of *T. catharinensis* as well as to examine the *in vitro* antioxidant and anticholinesterase activity of the extract and its main fractions and to relate biological activities to the identified compounds. 

## 2. Materials and Methods

Twenty samples of *Tabernaemontana catharinensis *were collected in Santo Angelo, RS, Brazil (28°27′59′′S, 54°29′37′′W) in November 2008. The plants were identified by professor Ronaldo A. Wasum and deposited in the herbarium of Universidade de Caxias do Sul (HUCS 34038–34057/guia 1669). 

After the removal of inflorescences, the terminal regions of aerial parts (leaves and branches) were dried in a greenhouse with forced air circulation at a temperature of 30°C for 4 days. The dry material was grinded in a Willey TE 650 grinder mill and stored in container protected from light for subsequent analysis.

### 2.1. Methods of Extraction and Purification

The triturated vegetal material underwent extraction with a Soxhlet apparatus using ethanol as extraction solvent (10 mL ethanol/g) for 12 hours at a temperature of nearly 70°C. The ethanolic extract was concentrated in a rotary evaporator at reduced pressure until complete removal of the solvent. 

**Figure 1:**
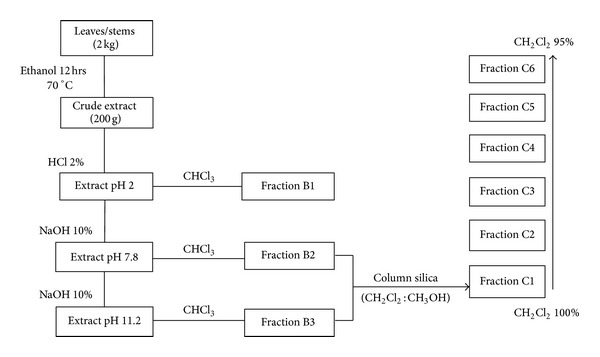
Diagram of fractionation of *Tabernaemontana catharinensis* extract.

According to the methodology described by Guida et al. [11], 150 mL of hydrochloric acid at 2% were added to each 10 grams of dry extract and extracted with 200 mL of chloroform (fraction named B1). The acid aqueous phase was alkalinized with sodium hydroxide 10% until pH 7.8 and extracted with 200 mL of chloroform (fraction B2). The aqueous phase was alkalinized until pH 11.2 with solution of sodium hydroxide 10% and extracted with 200 mL of chloroform (fraction B3) (Figure 1). 

Fractions B1, B2, and B3 were monitored by thin-layer chromatography (TLC) using aluminium chromatoplates precoated with silica gel F_254_ 0.2 mm (Merck) with eluent system CH_2_Cl_2_ : MeOH (93 : 7). Bands were visualized under ultraviolet light (254 and 365 nm) after staining with Dragendorff's reagent.

Fractions B2 and B3 had similar chromatographic profiles by TLC and were gathered. The mixture of these fractions underwent column chromatography using Merck silica gel (0.06–0.2) as a support and dichloromethane, dichloromethane with increasing percentages of methanol and methanol as eluent, according to Gower et al. [12]. A total of 14 fractions of 100ml each were collected and monitored by TLC, using the same procedure previously described and gathered according to the similarities observed, resulting in 6 fractions (C1–C6).

### 2.2. Identification of Alkaloids by Mass Spectrometry

The extract and fractions of *T. catharinensis *were dissolved in a solution of 50% (v/v) chromatographic grade acetonitrile (Tedia, Fairfield, OH, USA), acidified with 0.1% formic acid. The solutions were individually infused directly into the electrospray ionization (ESI) source by means of a syringe pump (Harvard Apparatus) at a flow rate of 10 *μ*L min^−1^. Electrospray ionization mass spectrometry (ESI(+)-MS) and tandem ESI(+)-MS/MS were acquired using a hybrid high resolution and high accuracy (5 *μ*L/L) Orbitrap XL mass spectrometer (Thermo Fisher Scientific) under the following conditions: capillary and cone voltages were set to +3500 V and +40 V, respectively, with a desolvation temperature of 100°C. For ESI(+)-MS/MS, the energy for the collision-induced dissociations (CID) was optimized for each component. Diagnostic ions in different fractions were identified by the comparison of their ESI(+)-MS/MS dissociation patterns with those of compounds identified in previous studies. For data acquisition and processing, Xcalibur software (Thermo Fisher Scientific) was used. The data were collected in the *m/z* range of 70–700, providing the resolution of 50,000 (FWHM) at *m/z* 200. No important ions were observed below *m/z* 200 or above *m*/*z* 650.

### 2.3. Determination of Antioxidant Activity

The antioxidant activity was measured by 2,2-diphenyl-1-picrylhydrazyl radical (DPPH^●^) scavenging activity [13]. Extract and fractions (B1–B3, C1–C6) of *T. catharinensis *(200 *μ*L) were added to Tris-HCl buffer 100 mM (pH 7.4, 800 *μ*L) and 1 mL DPPH^●^ (500 *μ*M dissolved in ethanol). The tubes were stored in the dark at room temperature for 20 min and absorbance was measured at 517 nm. Samples, blanks, and positive controls (ascorbic acid) were assessed in triplicate. Results were expressed as IC_50_, that is, the amount of the extract needed to scavenge 50% of DPPH^●^. 

### 2.4. Acetylcholinesterase Inhibitory Activity (AChE) and Evaluation of Kinetic Parameters

Acetylcholinesterase inhibitory activity was determined from the assay described by Ellman et al. [14]. Acetylthiocholine iodide (ATCI) and 5,5′dithiobis-(2-nitrobenzoic acid) (DTNB) were used as solvent and reagent for activity determination. Experiments used acetylcholinesterase from electric eel (Electrophorus electricus), Sigma C2888. In 96-well microplates, the reaction mixture containing 185 *μ*L of sodium phosphate buffer (concentration 0.1 M) pH 8.0, 5 *μ*L of DTNB (concentration 0.3 mM), 5 *μ*L of ATCI (concentration 0.5 Mm), and 5 *μ*L of solution were stored at a temperature of 25°C. Hydrolysis of ATCI initiated with the addition of 20 *μ*L of enzyme suspension (concentration 0.032 U/mL) and then microplates were read at 405 nm every 30 s for 2 min. Samples, blanks, and positive controls (galantamine) were assessed in triplicate. The results were expressed as IC_50_, that is, the concentration required to inhibit 50% enzymatic activity.

The same methodology was used to evaluate kinetic parameters, except for DTNB content (concentration 1 mM) and multiple ATCI concentrations (0.5, 0.6, 0.9, 1.35, 1.8, and 3 mM) [15, 16]. Negative and positive controls (galantamine 0.2 and 1 *μ*g/mL) and fraction C6 (0.25, 1 and 100 *μ*g/mL) were assessed in triplicate. The values obtained were graphed according to the Michaelis-Menten and Lineweaver-Burk models.

### 2.5. Statistical Analysis

Results underwent statistical analysis by analysis of variance and Tukey's post test (*P* ≤ 0.05) using SPSS16.0 software for Windows (SPSS Inc., Chicago, IL, USA).

## 3. Results and Discussion

**Figure 2:**
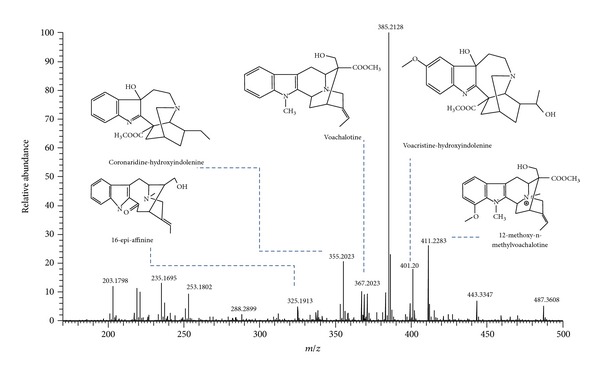
Electrospray positive spectra of *T. catharinensis* extract and structures of identified alkaloids compounds.

ESI(+)-MS analysis of the raw extract of *T. catharinensis* revealed the presence of chemicals with molecular weight ranging from 203 to 487 (Figure 2). The results obtained in this study, also considering previous reports of the genus *Tabernaemontana* [17, 20], indicated the presence of the following alkaloids: 16-epi-affinine (*m/z *325.1911), coronaridine-hydroxyindolenine (*m/z* 355.2019), voachalotine (*m/z *367.2020), voacristine-hydroxyindolenine (*m/z *401.2072), 12-methoxy-n-methyl-voachalotine (*m/z *411.2280), and of an unidentified compound with *m/z* 385.2125, whose fragmentation profile suggested a derivative of voacristine or voacangine. 

**Figure 3:**
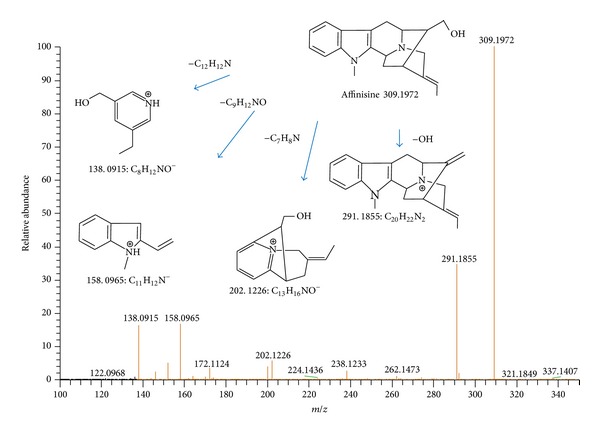
ESI(+)-MS/MS of affinisine.

Table 1 lists the compounds identified in the raw extract of *T. catharinensis* by ESI(+)-MS as well as the analyses of the main precursor ions by ESI(+)-MS/MS. Indole alkaloids were characterized by the important ability to stabilize positive charges, provided by the nitrogen atom and by the presence of carboxyl groups that can be ruptured in situations of ionization [23]. These characteristics can be evidenced in Figure 3, in which, through ion isolation and fragmentations, it was possible to confirm the chemical structure of the affinisine compound.

**Figure 4:**
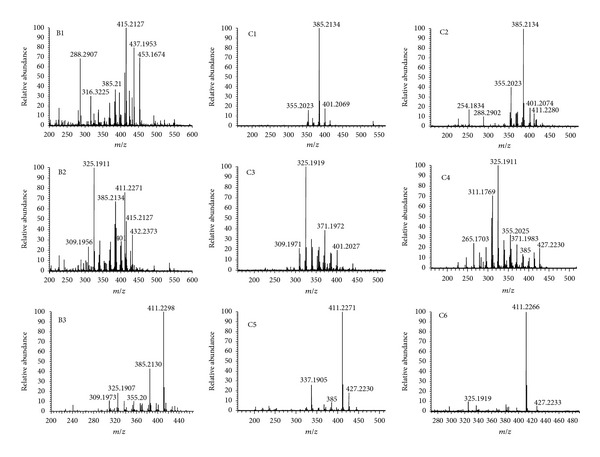
2 Electrospray positive spectra of fractions B1–B3, C1–C6.

The fractions obtained from the dry extract were analyzed by ESI(+)-MS (Figure 4). Fractions B2 and B3 (0.4% of the initial material) had similar chromatographic profiles by TLC and showed significant differences in the spectrum obtained by ESI(+)-MS. Fractions C1 and C2 were characterized by the presence of the possible derivative of voacristine or voacangine (*m/z* 385.2134). On the other hand, fractions C3 and C4 had a higher peak corresponding to 16-epi-affinine (*m/z* 325.1911) as well as an unidentified compound with m/z 311.1769. Fractions C5 and C6, in turn, had 12-methoxy-n-methyl-voachalotine (*m/z* 411.2171) as the most intense peak in the spectrum.

Of the compounds identified in the raw extract of *T. catharinensis*, affinisine was previously described in *T. catharinensis* extracts [24] as well as in extracts from *T. buchtieni* [25], *T. fuchsiaefolia* [17], and *T. histrix* [9]. In turn, 16-epi-affinine was identified in *T. catharinensis*, *T. latea*, *T. pachysiphon* and *T. psychotrifolia* [18], and *T. fuchsiaefolia* [22]. *Vobasine* is present in several species of the genus *Tabernaemontana*, including *T. catharinensis* [21]. *Coronaridine-hydroxyindolenine*, in turn, was identified in *T. catharinensis* extracts [20] and was also found in *T. divaricata* [26], *T. buchtieni* [25], and *T. heyneana* [27]. *Voachalotine* and 12-methoxy-n-methyl-voachalotine were detected in extracts from *T. catharinensis* [20] and *T. fuchsiaefolia* [17], among other species of the genus. Voacristine-hydroxyindolenine, in turn, was described in *T. apoda* and *T. dichotoma* [21], in *T. buchtieni* [25], and also in *T. heyneana* [27].

Thus, generally speaking, the compounds identified in the raw ethanolic extract of *T. catharinensis* are consistent with previous studies on this vegetal species. However, the major indole alkaloids, voacangine, and coronaridine, described in branch extracts obtained by supercritical CO_2_ [28, 29] and in leaf ethanolic extracts [29], were not detected in the present research. The absence of these compounds may be attributed to environmental and genetic differences in the evaluated material, or to the analysis methods used, since these authors employed liquid chromatographic/mass spectrometric (LC/MS) and gas chromatography/mass spectrometry (GC/MS), respectively. It is worth highlighting that coronaridine-hydroxyindolenine and a possible derivative of voacristine or voacangine were detected in the raw extract and in some of its fractions.

The extract of *T. catharinensis* and its fractions were evaluated with regard to their DPPH^●^ scavenging activity, a method routinely used and recognized due to its practicality and rapidity [30, 31]. According to the results shown in Table 2, the raw extract exhibited DPPH^●^ scavenging activity with IC_50_ of 313.46 *μ*g/mL. Fraction B1, which had low content of alkaloids according to the chromatographic analysis, exhibited lower antioxidant activity than the raw extract, with IC_50_ of 1.590.00 *μ*g/mL. 

Fractions C1 and C2, which had similar spectra, with the presence of a possible derivative of voacristine or voacangine, showed a significant difference in their antioxidant activities. We suggest that the higher activity observed in fraction C2 may be attributed to other unidentified compounds or to its content of coronaridine-hydroxyindolenine (Figure 4). On the other hand, fractions C3 and C4, characterized by the presence of 16-epi-affinine and of an unidentified compound with *m/z* 311.1769, exhibited similar antioxidant activity, close to that of fractions B2 and B3. The lower antioxidant activity among the fractions obtained by chromatographic separation was observed in fractions C5 and C6, which were characterized by the presence of 12-methoxy-n-methyl-voachalotine.

The antioxidant activity of the raw extract from the aerial parts of *T. catharinensis *was lower than that observed in root ethanolic extracts of this species (IC_50_ = 100 *μ*g/mL) [32] but higher than that identified in ethanolic extracts of *T. heyneana *leaves (IC_50_ = 537 *μ*g/mL) [33].

Indole alkaloids have been previously described as exhibiting antioxidant activity [32, 34]. Studies showed that reactive oxygen species uptake activity in indole derivatives could be related to the exceptional redox property of the indole ring, which is especially conferred by the indole nitrogen [35]. Among the indole alkaloids present in *Tabernaemontana*, antioxidant activity has been identified for isovoacristine-hydroxyindolenine and voacangine [32]. On the other hand, variations in the content of coronaridine and voacangine in *T. catharinensis *extracts did not change *in vitro* antioxidant potential [8]. 

The results for the inhibitory activity of the extract of *T. catharinensis* and its fractions on acetylcholinesterase (AChE) enzyme using Ellman's method [14] can also be observed in Table 2. The raw extract had the ability of inhibiting AChE enzyme (IC_50_ = 261.55 *μ*g/mL). This antiacetylcholinesterase activity was previously reported in the ethanolic extract of *Tabernaemontana divaricata* roots (IC_50_ = 2.56 *μ*g/mL), in which there has been also reports of selective and reversible enzyme inhibition [36].

Fractions B2 and B3, as well as samples obtained by column fractionation, showed higher values for AChE enzyme inhibition when compared to the value observed for the raw extract. Fractions C5 and C6 were those that obtained better results for AChE inhibition, with IC_50_ of 2.50 *μ*g/mL and 2.10 *μ*g/mL, respectively. On the other hand, fraction B1 exhibited low anticholinesterase activity (IC_50_ = 458.40 *μ*g/mL). These results corroborate previous reports that mentioned the anticholinesterase potential of alkaloids found in the genus *Tabernaemontana* and the possibility of using these compounds for studies involving the treatment of neurodegenerative diseases [9, 24].

Significant enzyme inhibition activities were observed in fractions C3 (IC_50_ 7.71 *μ*g/mL), C4 (IC_50_ 8.34 *μ*g/mL), and B3 (IC_50_ 9.0 *μ*g/mL). These fractions were characterized by the presence of the compounds affinisine, 16-epi-affinine, vobasine, and coronaridine-hydroxyindolenine. In previous reports, affinine and affinisine inhibited AChE enzyme [9, 24]. On the other hand, vobasine and coronaridine-hydroxyindolenine showed low inhibition results at the concentrations evaluated by Zhan et al. [37]. 

Recent studies related the molecular structure of indole alkaloids to AChE inhibitory capacity, based on compounds similar to coronaridine. It was observed that structures with hydrophobic substituents or electron-donor substituents exhibited higher inhibitory activity when compared to their hydroxylated derivatives [37]. However, these attributes were not observed with isovoacangine and its hydroxilated derivative [9]. Representatives of the Corinantea class such as 12-methoxy-n-methyl-voachalotine, were characterized by exhibiting antiacetylcholinesterase activity [24]. It is worth stressing that this case requires further clarification when relating structure to activity potential, taking into account that n-methyl-voachalotine showed negative results for enzyme inhibition in previous reports [9]. 

**Figure 5:**
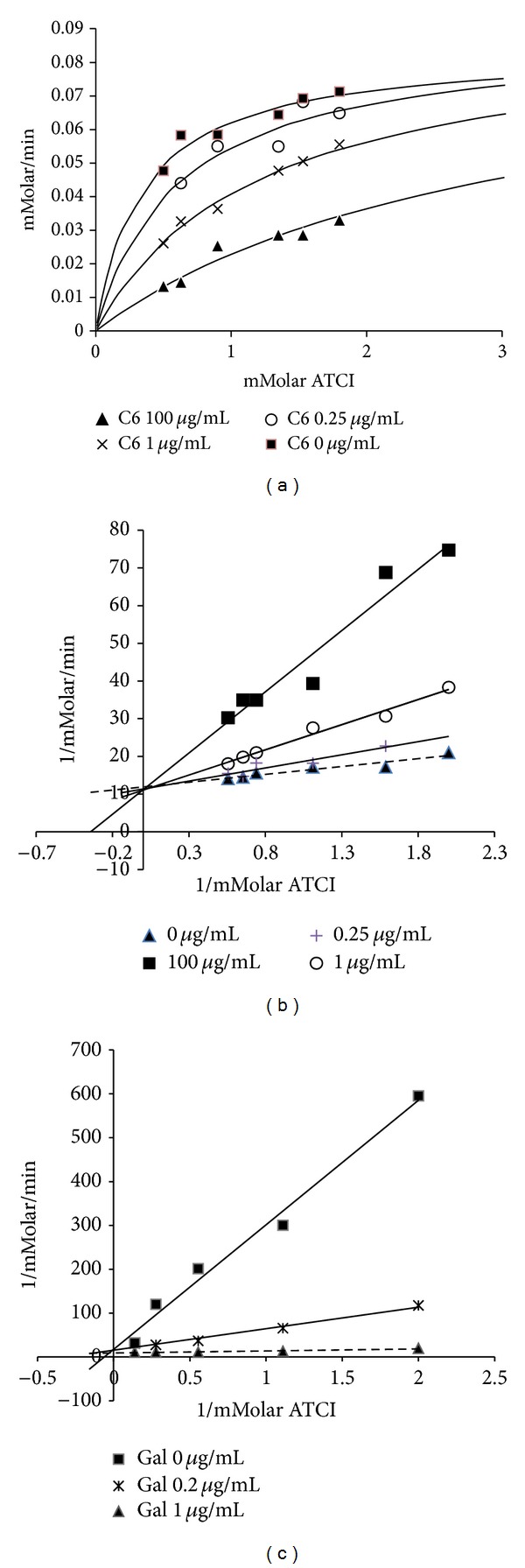
Kinetic study of inhibition of acetylcholinesterase by C6 fraction and galantamine (standard acetylcholinesterase inhibitor). (a) Michaelis-Menten kinetic for C6 fraction, (b) double reciprocal (Lineweaver Burk) plot for C6 fraction, and (c) double reciprocal (Line-weaver Burk) plot for galantamine.

In an attempt of understanding inhibition results for fraction C6, kinetic studies were conducted, which may suggest possible models of interaction between AChE and its inhibitors [38]. It is known so far that AChE, an enzyme belonging to the family of *αβ* hydrolases, has two main binding sites: an active site (ACS) and an anionic site (PAS) [39].  Figure 5 shows the models of enzyme kinetics and Michaelis-Menten as well as the linear model described by Lineweaver-Burk with different inhibitor concentrations (fraction C6). 

According to the graph that associated varied substrate concentrations (1/mMolar ATCI) with velocities of product formation (1/mMolar/min) (Figure 5(b)), it is possible to find that high substrate concentrations displace the balance of the reaction and favor the formation of the enzyme-substrate complex, thus reducing the likelihood of the inhibitor (C6) to bond to the enzyme active site. This characteristic can be observed when considering the intersection between the reaction with and without the inhibitor on the *y*-axis. This behavior is typical of competitive inhibitors such as galantamine (Figure 5(c)), as described by Khan et al. [38]. In addition, the maximum velocity of product formation obtained in the absence or presence of C6 inhibitor at different concentrations remained constant (*V*_max⁡_ = 0.079 mMolar/min), and different *K*_*m*_ values were observed in the absence of the inhibitor (*K*_*m*_ = 0.28 mMolar ATCI) and in the presence of C6 at a 1 *μ*g/mL concentration (*K*_*m*_ = 1.20 mMolar ATCI). This is the first report of a kinetic study involving the model of interaction between AChE and fractions containing indole alkaloids found in *Tabernaemontana*, suggesting competitive inhibitory action. 

Structures with quaternary nitrogen exhibit significant binding activity to aromatic wastes at the AChE active site [40], which justifies the activity of the fraction C6 according to the structural characteristics of the compound 12-methoxy-n-methyl-voachalotine, the most intense peak in C6 spectrum. However, it is worth emphasizing the need of purifying this compound in order to confirm these results. Even so, there is the possibility that the alkaloids described in this paper exhibit also bonding connection to subtypes of receptors, similar to other compounds already evaluated [41]; therefore, *in vitro* studies are also necessary in order to examine these possible mechanisms.

 Given the potential of the extracts of *T. catharinensis *and their compounds, further investigations should be conducted with the purpose of purifying, identifying, and confirming their biological properties, having in view their therapeutic use.

## Figures and Tables

**Table 1:** Chemical composition of *T. catharinensis *extract by ESI(+)-MS e ESI(+)-MS/MS.

Entry	Precursor ion *m*/*z*	Fragmentation ms/ms (%) [MS2] {MS3}	Identification	Elem. comp.	Diff. ppm	Fragmentation pathways	References
1	309.1963	291.1864(35); 202.1232(5); 158.0968(17); 138.0917(16)	Affinisine	C_20_H_24_N_2_O	0.646	291.1864[M-OH]; 202.1232[M-C_7_H_8_N]; 158.0968[M-C_9_H_12_NO]	[9, 17]

2	325.1918	308.1891(15); 307.1814(13) [289.1706(4); 265.1700(100) {234.1281(11); 122.0966(8)}176.1071(5)]; 265.1706(5); 152.1074(12)	16-epi-Affinine	C_20_H_24_N_2_O_2_	2.460	308.1891[M-OH]; 265.1700[M-C_2_H_3_O_2_]; 152.1074[M-C_11_H_10_NO]	[18]

3	353.1866	322.1443(9); 321.1606(33), [293.1654(65), 275.1548(22) 264.1388(100), 221.1078(24), 211.0870(65), 183.0921(98)] 290.1184(6)	Vobasine	C_21_H_24_N_2_O_3_	1.981	322.1443[M-C_2_H_6_]; 293.1654[M-C_2_H_4_O_2_] 275.1548[M-C_2_H_6_O_3_]; 264.1388[M-C_3_H_7_NO_2_]	[19]

4	355.2023	337.1920(100) [305.1656(60); 277.1706(21); 216.1024(8); 144.0811(9)]; 323.1762(7); 305.1657(60) [290.1418(22); 277.1704(100) {207.0921(10)}; 235.0869(67); 184.0760(36); 174.0916(33)]	Coronaridine-hydroxyindolenine	C_21_H_26_N_2_O_3_	1.970	337.1920[M-OH]; 323.1762[M-CH_3_O] 305.1657[M-CH_4_O_2_]; 277.1704[M-C_2_H_5_O_3_]	[20]

5	367.2023	337.1921(100) [305.1655(28); 172.1125(16); 158.0967(34)]; 335.1765(7); 305.1658(23); 172.1126(31); 166.0868(5); 158.0969(7)	Voachalotine	C_22_H_26_N_2_O_3_	1.906	337.1921[M-CH_3_OH]; 305.1655[M-C_2_H_4_O_2_] 172.1125[M-C_12_H_12_NO_3_]; 158.0969[M-C_11_H_15_NO_3_]	[21]

6	385.2128	367.2024(100), [335.1761(100), {307.1812(40), 265.0977(21), 214.0867(9), 174.0917(15)}; 307.1810(100) 246.1130(19); 174.0918(10)]; 335.1765(20); 307.1815(7)	Derivative of voacristine or voacangine	n.d			

7	401.2075	383.1975(100) [365.1867(100); {333.1604(20); 201.1026(26)} 351.1711(21); 337.1553(21); 201.0976(23)]; 365.1867(62); [351.1711(21); 337.1553(21); 333.1605(7); 323.1760(8); 201.1027(23)]; 351.1715(7); 201.1029(6)	Voacristine- hydroxyindolenine	C_22_H_28_N_2_O_5_	1.246	383.1975[M-OH]; 365.1867[M-O_2_H_4_] 351.1711[M-CH_7_O_2_]; 333.1604[M-CH_7_O_3_] 201.1027[M-C_10_H_15_O_4_]	[17]

8	411.2283	381.2181(100); 349.1917(100); [334.1681(16); 321.1966(10); 317.1654(15); 266.1544(35); {251.1309(21); 237.1151(44)}]; 200.1076(100); [185.0839(14); 169.0890(12)]; 180.1024(25)	12-Methoxy-n-methyl-voachalotine	C_24_H_31_N_2_O_4_	1.215	381.2181[M-CH_4_O]; 349.1917[M-C_2_H_6_O_2_] 334.1681[M-C_3_H_8_O_2_]; 321.1966[M-C_3_H_7_O_3_] 200.1076[M-C_11_H_15_NO_3_]	[22]

9	415.2126		ni				

ni: not identificated.

**Table 2:** Antioxidant and anticholinesterasic activity of *T. catharinensis *extract and fractions.

Samples	DPPH-IC_50_ (*μ*g/mL)	AChE inhibition-IC_50_ (*μ*g/mL)
Extract	313.46 ± 0.5^b^	261.55 ± 9.1^b^
Organic/aqueus fractions		
B1	1590.00 ± 1.4^a^	458.40 ± 8.2^a^
B2	60.75 ± 0.5^e^	18.35 ± 1.4^d^
B3	67.28 ± 0.6^e^	9.0 ± 0.4^e^
c. column fractions		
C1	94.92 ± 0.05^d^	17.35 ± 1.4^d^
C2	37.18 ± 0.1^f^	91.22 ± 7.1^c^
C3	74.69 ± 0.9^e^	7.71 ± 0.1^e^
C4	65.59 ± 0.3^e^	8.34 ± 0.6^e^
C5	230.25 ± 0.1^c^	2.50 ± 0.1^f^
C6	249.61 ± 0.9^c^	2.10 ± 0.1^f^
Ascorbic acid	20.13 ± 0.45^g^	—
Galantamine	—	0.2 ± 0.05^g^

Different letters correspond to values statistically different by analysis of variance (ANOVA) and Tukey's post hoc test, for *P* ≤ 0.05, for each activity.
